# Prevalence, distribution and evolutionary significance of the IS*629 *insertion element in the stepwise emergence of *Escherichia coli *O157:H7

**DOI:** 10.1186/1471-2180-11-133

**Published:** 2011-06-14

**Authors:** Lydia V Rump, Markus Fischer, Narjol Gonzalez-Escalona

**Affiliations:** 1 Division of Microbiology, Center for Food Safety and Applied Nutrition, Food and Drug Administration, 5100 Paint Branch Parkway, College Park, MD 20740, USA; 2 Institute of Food Chemistry, University of Hamburg, Hamburg, Germany

## Abstract

**Background:**

Insertion elements (IS) are known to play an important role in the evolution and genomic diversification of *Escherichia coli *O157:H7 lineages. In particular, IS*629 *has been found in multiple copies in the *E. coli *O157:H7 genome and is one of the most prevalent IS in this serotype. It was recently shown that the lack of O157 antigen expression in two O rough *E. coli *O157:H7 strains was due to IS*629 *insertions at 2 different locations in the *gne *gene that is essential for the O antigen biosynthesis.

**Results:**

The comparison of 4 *E. coli *O157:H7 genome and plasmid sequences showed numerous IS*629 *insertion sites, although not uniformly distributed among strains. Comparison of IS*629*s found in O157:H7 and O55:H7 showed the presence of at least three different IS*629 *sub-types. O157:H7 strains carry IS*629 *elements sub-type I and III whereby the ancestral O55:H7 carries sub-type II. Analysis of strains selected from various clonal groups defined on the *E. coli *O157:H7 stepwise evolution model showed that IS*629 *was not observed in sorbitol fermenting O157 (SFO157) clones that are on a divergent pathway in the emergence of O157:H7. This suggests that the absence of IS*629 *in SFO157 strains probably occurred during the divergence of this lineage, albeit it remains uncertain if it contributed, in part, to their divergence from other closely related strains.

**Conclusions:**

The highly variable genomic locations of IS*629 *in O157:H7 strains of the A6 clonal complex indicates that this insertion element probably played an important role in genome plasticity and in the divergence of O157:H7 lineages.

## Background

Enterohemorrhagic *Escherichia coli *(EHEC) of serotype O157:H7 has been implicated in foodborne illnesses worldwide. It frequently causes large outbreaks of severe enteric infections including bloody diarrhoea, hemorrhagic colitis (HC) and haemolytic uremic syndrome (HUS) [1,2]. This serotype constitutively expresses the somatic (O) 157 and flagellar (H) 7 antigens, thus, these traits are used extensively in clinical settings to identify this highly pathogenic serotype [1]. However some O157:H7 strains, although being genotipically O157 or H7 do not express either of those antigens [3,4]. According to the latest CDC report, *E. coli *O157:H7 infections affect thousands of people every year accounting for 0.7%, 4% and 1.5%, of illnesses, hospitalizations and deaths, respectively of the total U.S. foodborne diseases caused by all known foodborne pathogens [5].

Previously, we characterized two potentially pathogenic O *rough*:H7 strains that did not express the O157 antigen [4,6] but belonged to the most common O157:H7 clonal type. The O *rough *phenotype was found to be due to two independent IS*629 *insertions in the *gne *gene that encodes for an epimerase enzyme essential for synthesis of an oligosaccharide subunit in the O antigen. Of the IS elements identified in O157 strains, IS*629 *elements are the most prevalent in this serotype and have been confirmed to very actively transpose in O157 genomes [7]. The presence of O-rough strains of this serotype in food and clinical samples is of concern as they cannot be detected serologically in assays routinely used to test for O157:H7 [3].

The occurrence of other atypical O157:H7 strains due to IS*629 *insertions therefore, might be more common than anticipated. It is generally assumed that IS elements play important roles in bacterial genome evolution and in some cases are known contributors to adaptation and improved fitness [7]. The acquisition or loss of mobile genetic elements, like IS elements, may differ between strains of a particular bacterial species [8]. IS insertion and IS-mediated deletions have been shown to generate phenotypic diversity among closely related O157 strains [7]. It has been shown that O157 is a highly diverse group and a major factor that effects this diversity are prophages [7]. However, in addition to prophages, IS*629 *also appears to be a major contributor to genomic diversification of O157 strains. Therefore, it is questionable how much influence IS*629 *had on the evolution of O157:H7, or how much importance IS*629 *has to changes in virulence in this bacterium.

**Figure 1:**
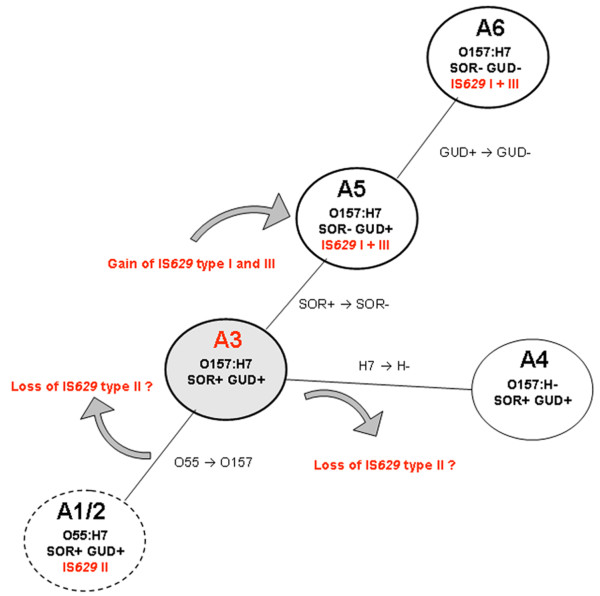
**Stepwise evolutionary model for *E. coli *O157:H7 from ancestral O55:H7 **[11]. In red letters are the possible events happening and where they occurred during the stepwise evolution. The circle in gray represents an intermediary A3 CC, which has not yet been isolated. SOR - sorbitol fermentation [if (+) fermenting, if (-) non-fermenting or slow fermenting]. GUD - β-D-glucuronidase activity.

It has been proposed in an evolutionary model previously that highly pathogenic enterohemorrhagic *E. coli *(EHEC) O157:H7 arose from its ancestor enteropathogenic *E. coli *(EPEC) O55:H7 (SOR+ and GUD+) through sequential acquisition of virulence, phenotypic traits, and serotypic change (A1(*stx*^-^)/A2(*stx*2) in Figure 1A) [9-11]. After the somatic antigen change from O55 to O157 gave rise to an intermediary (A3) which has not yet been isolated, two separate O157 clonal complexes evolved, splitting into two diverged clonal groups. One of these groups was composed of sorbitol fermenting (SF) non-motile O157:NM strains containing plasmid pSFO157 (A4) (SOR+, GUD+). The other was composed of non-sorbitol fermenting (NSF) O157:H7 strains containing plasmid pO157 (A5) (SOR-, GUD+). The latter, by a mutational inactivation of the *uidA *gene, lost its β-glucuronidase activity which is the most typical O157:H7 phenotype at present (A6) [11]. These A6 strains have spread geographically into disparate locales and now account for most of the diseases caused by EHEC [12].

IS*629 *seems to play an important role in the diversification of closely related strains, specifically O157:H7 [7]. In the present study, we examined the prevalence of IS*629 *in a panel of *E. coli *strains, including ancestral and atypical strains associated with the stepwise emergence of *E. coli *O157:H7 to determine the prevalence of IS*629 *and its impact on the transitional steps that gave rise to today's highly pathogenic *E. coli *O157:H7.

## Results

### IS*629 *prevalence in *E. coli *O157:H7 genomes

The IS*629 *sequence, recently found to be inserted into the *gne *gene in *E. coli *O *rough*:H7 (MA6 and CB7326) [4,13], was used for a BLAST analysis of the genomes of 4 *E. coli *O157:H7 strains belonging to A6 CC (EDL933, Sakai, EC4115 and TW14359) and one O55:H7 strain (CB9615) (Additional file 1, Table S1). The BLAST analysis for IS*629 *showed the presence of between 22 and 25 copies in each strain along with their corresponding plasmid (Table 1). Strains Sakai and EDL933 shared 13 of those IS*629 *on the chromosome and three on their pO157 plasmids. Strains EC4115 and TW14359 had 17 IS*629 *on the chromosome and four on their pO157 plasmid in common. The analysis of the recently released *E. coli *O55:H7 genome strain CB9615 [14] allowed for identification of one IS*629 *with an internal 86 bp deletion on the chromosome and an IS*629 *in its corresponding pO55 plasmid. Neither the O55 genomic (located on the chromosome backbone) nor the pO55 plasmid IS*629 *insertion sites were present in other O157:H7 strains. The absence of the pO55 IS*629 *insertion site in O157:H7 strains was expected since they do not carry the pO55 plasmid. However, lack of the genomic O55 IS*629 *insertion site in O157:H7 strains is interesting as these strains are known to be closely related [14]. Contrary to what was observed for plasmids pO157 and pO55, IS*629 *was absent in plasmid pSFO157 (*E. coli *O157:H- strain 439-89). However, a 66 bp sequence identical to IS*629 *was observed in the plasmid which could be a remnant of IS*629*. No genomic sequence is available for an O157:H- strain at this time, thus, this strain could not be investigated for the presence of IS*629*.

**Table 1:** Prevalence of IS*629 *elements in each strain (chromosomes and plasmids) and number of shared IS*629*

	Strain	Serotype	IS*629 *Sites	In common with strain				
				Sakai	EDL933	EC4115	TW14359	CB9615
**Chromosomes**								
	**Sakai**	O157:H7	19	-	13	9	9	0
	**EDL933**	O157:H7	21	13	-	6	6	0
	**EC4115**	O157:H7	19	9	6	-	17	0
	**TW14359**	O157:H7	21	9	6	17	-	0
	**CB9615**	O55:H7	1	0	0	0	0	-

**Plasmids**				**pO157 Sakai**	**pO157 EDL933**	**pO157 EC4115**	**pO157 TW14359**	**pO55 CB9615**
	**pO157 Sakai**	O157:H7	3	-	3	3	3	0
	**pO157 EDL933**	O157:H7	3	3	-	3	3	0
	**pO157 EC4115**	O157:H7	4	3	3	-	4	0
	**pO157 TW14359**	O157:H7	4	3	3	4	-	0
	**pSFO157**	O157:H-	0	0	0	0	0	0
	**pO55 CB9615**	O55:H7	1 tr*	0	0	0	0	-

tr* - truncated.

### IS*629 *target site specificity ("hot spots") on chromosomes and plasmids of four *E. coli *O157:H7 strains

The majority of IS*629 *elements were located on prophages or prophage-like elements (62%) ("strain-specific-loops", S-loops in Sakai [15]). 28% of IS*629 *locations were found on the well-conserved 4.1-Mb sequence widely regarded as the *E. coli *chromosome backbone (*E. coli *K-12 orthologous segment) [15] and 10% were located on the pO157 plasmid. In total, we observed 47 different IS*629 *insertion sites (containing complete or partial IS*629*) in the four *E. coli *chromosomes and plasmids by "in silico" analysis (Additional file 2, Table S2). Seven of 47 IS*629 *insertion were shared among the 4 diverged strains which suggest that they were also present in a common ancestor.

### IS*629 *presence in strains belonging to the stepwise model of emergence of *E. coli *O157:H7

**Figure 2:**
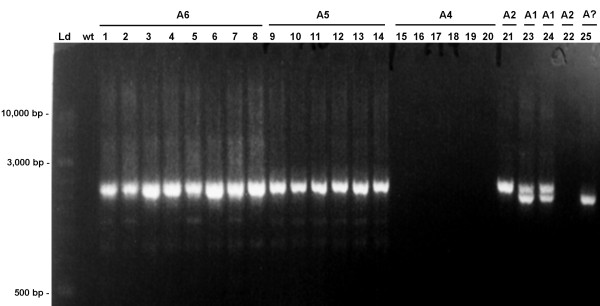
**Gel-electrophoresis of the PCR products for IS*629 *presence in strains belonging to the stepwise model of emergence of *E. coli *O157:H7**. Lanes: Ld, molecular weight ladder (Gene Ruler); wt, Blank; 1 - 25, strains numbered according to Table 2. A1-A6, Clonal complexes, A?, CC unknown.

A total of 27 *E. coli *strains (Table 2) belonging to the stepwise model proposed by Feng et al. (1998) were examined by PCR for the presence of IS*629 *using specific primers [16]. Every strain of clonal complex (CC) A6, A5, A2 and A1 carried IS*629*, except strain 3256-97 belonging to the ancestral CC A2 (Figure 1). Strikingly, however, was the observation that IS*629 *was absent in the SFO157 strains belonging to the closely related CC A4 (Figure 2). Whole genome analysis of two A4 strains (493-89 accession no. AETY00000000 and H2687 accession no. AETZ00000000) confirmed the absence of this specific IS element in SFO157 strains [17]. On the other hand, O55:H7 strain 3256-97 (AEUA00000000) carried a truncated IS*629 *version missing the target area for the reverse primer (IS*629*-insideR) located in ORFB, explaining the lack of IS*629 *by PCR [17]. Additionally, strains USDA5905 (A2) and TB182A (A1) as well as strain LSU-61 (A?) appear to harbor a truncated IS*629 *which could indicate the presence of genomic IS*629 *found in the O55 strain CB9615. However, since no additional ancestral strains were available for analysis, the distribution of IS*629 *in these groups is at present inconclusive.

**Table 2:** Serotype, sequence type, characteristics and isolation information of strains of *E. coli *used in this study

No.	Name	Other name	Serotype	*stx*	Special characteristics			ST	CC	Source	Year	Reference
					GUD	SOR	plasmid					
1	Sakai	BAA 460	O157:H7	1, 2	-	-	pO157	66	A6	Japan	1996	NC_002695
2	EDL 933	700927	O157:H7	1,2	-	-	pO157	66		USA	1982	AE005174
3	EC 4115		O157:H7	1, 2	-	-	pO157	66		USA	2006	NC_011353
4	TW 14359		O157:H7	1, 2	-	-	pO157	66		USA	2006	CP001368
5	EDL 931	35150	O157:H7	1, 2	-	-	pO157	66				[26]
6	MA6		O157:H7	2	-	-	pO157	66		Malaysia	1998	[6]
7	550654		O157:H7	2	-	-	pO157	66		USA	2009	
8	FDA 413		O157:H7	2	-	-	pO157	66				[10]

9	G5101		O157:H7	1, 2	+	-	pO157	65	A5	USA	1995	[11]
10	1628		O157:H7	1, 2	+	-	pO157	65				[32]
11	1659		O157:H7	1, 2	+	-	pO157	65				[11]
12	EC 97144	TW 10707	O157:H7	1, 2	+	+	pO157	65		Japan	1997	[33]
13	EC 96038	TW 10201	O157:H7	1, 2	+	+	pO157	65				[11]
14	EC 96012	TW 10189	O157:H7	1, 2	+	+	pO157	65				[11]

15	493-89		O157:H-	2	+	+	pSFO157	75	A4	Germany	1989	[11]
16	5412-89		O157:H-	2	+	+	pSFO157	75		Germany	1989	[34]
17	H56929	TW 09159	O157:H-	2	+	+	pSFO157	76		Finland	1999	[11]
18	H56909	TW 09162	O157:H-	2	+	+	pSFO157	76		Finland	1999	[11]
19	H 1085c		O157:H-	2	+	+	pSFO157	76		Scotland	2003	[11]
20	H 2687		O157:H-	2	+	+	pSFO157	76		Scotland	2003	[11]

21	3256-97	TW 07815	O55:H7	2	+	+	?	73	A2	USA	1997	[11]
22	USDA 5905		O55:H7	2	+	+	?	73		USA	1994	[26]

23	TB 182A	TW 04062	O55:H7	-	+	+	?	73	A1	USA	1991	[11]
24	DEC5A		O55:H7	-	+	+	?	73				[11]

25	LSU-61		O157:H7	-	+	+	?	237	?	USA	2001	NC_002695

26	Sakai PF		O157:H7	1, 2	-	-	pO157	66	A6	Japan	1996	AE005174
27	43895	CDC EDL 933	O157:H7	1,2	-	-	pO157	69	A6	USA	1982	

*stx *- shiga toxin gene, GUD - β-glucuronidase activity, SOR - sorbitol fermentation, ST - sequence type as determined by a combination of seven genes http://www.shigatox.net/stec/cgi-bin/index, CC - Clonal complex [11], ? - Unknown. Sakai PF and 43895 are strain derived after numerous subculture passages from the original Sakai and EDL933 strains, respectively.

### IS*629 *distribution in strains belonging to the stepwise model of emergence of *E. coli *O157:H7

We successfully PCR amplified 38 of the 47 observed IS*629 *insertion sites in the 27 O157:H7 strains analyzed (Additional file 3, Table S2). We determined presence or absence of an IS*629 *element as well as the IS*629 *target site in each strain (Additional file 1, Figure S1). In accordance with the previous finding of total absence of IS*629 *in SFO157, none of the A4 CC strains harbored an IS*629 *in any of the known IS*629 *insertion sites. Likewise, it was observed for A1 and A2 CC strains, indicating that the previously detected IS*629 *must be located in some other region of the chromosome. In A5 CC strains, only 3 of the 38 (7%) IS*629 *insertion sites harbored an IS*629 *(Table 3). Those sites were located on the prophage Sp12, the prophage-like element SpLE1, and on the chromosomal backbone. Interestingly one of the A5 CC strains (strain 1659) did not share any of the known sites harboring IS*629*. The A6 CC strains shared between 6 (16%) and 21 (55%) IS*629 *insertions in the known sites and two of them (IS.15: Sp14 and IS.41: pO157) were present in all A6 CC strains. IS*629 *prevalence in the A6 strains and the distribution amongst Sp, SpLE, backbone and the pO157 plasmids did not show any specific pattern, however it appears that IS*629 *transposes actively in the A6 CC.

**Table 3:** IS*629 *element presence/absence in CC strains from the O157:H7 stepwise evolutionary model

					A6								A5					A4				A2		A1		A?		A6
**NR**	**Phage** **Or** **backbone**	**1**	**2**	**3**	**4**	**5**	**6**	**7**	**8**	**9**	**10**	**11**	**12**	**13**	**14**	**15**	**16**	**17**	**18**	**19**	**20**	**21**	**22**	**23**	**24**	**25**	**26**	**27**

IS.1	Sp 4	ND	ND	ND	ND	ND	ND	ND	ND	ND	ND	ND	ND	ND	ND	ND	ND	ND	ND	ND	ND	ND	ND	ND	ND	ND	ND	ND
IS.2	Sp 4	ND	ND	ND	ND	ND	ND	ND	ND	ND	ND	ND	ND	ND	ND	ND	ND	ND	ND	ND	ND	ND	ND	ND	ND	ND	ND	ND
IS. 3	Sp 5 stx2	+	-	-	-	-	-	-	-	-	-	-	-	-	-	-	-	-	-	-	-	-	-	-	-	-	+	-
IS. 4	SpLE 1	-	+	-	-	-	-	+	-	-	-	-	-	-	-	-	-	-	-	-	-	-	-	-	-	-	-	-
IS. 5	SpLE 1	+	+	+	+	+	-	+	-	-	-	-	-	-	-	-	-	-	-	-	-	-	-	-	-	-	+	+
IS. 6	SpLE 1	-	+	-	-	-	-	+	-	-	-	-	-	-	-	-	-	-	-	-	-	-	-	-	-	-	-	-
IS. 7	SpLE 1	+	+	+	+	+	-	+	+	-	-	-	-	-	-	-	-	-	-	-	-	-	-	-	-	-	+	+
IS. 8	Sp 8	+	-	+	-	+	-	+	-	-	-	-	-	-	-	-	-	-	-	-	-	-	-	-	-	-	+	-
IS. 9	Sp 8	-	+	-	-	-	-	-	-	-	-	-	-	-	-	-	-	-	-	-	-	-	-	-	-	-	-	+
IS. 10	back	+	+	-	-	+	-	+	+	-	-	-	-	-	-	-	-	-	-	-	-	-	-	-	-	-	+	+
IS. 11	back	+	+	-	-	+	-	-	-	-	-	-	-	-	-	-	-	-	-	-	-	-	-	-	-	-	+	+
IS. 12	Sp 12	+	-	-	-	-	-	-	-	-	-	-	+	+	+	-	-	-	-	-	-	-	-	-	-	-	+	-
IS. 13	back	+	+	+	+	+	+	+	-	-	-	-	-	-	-	-	-	-	-	-	-	-	-	-	-	-	+	+
IS. 14	Sp 13	+	+	+	+	+	+	-	+	-	-	-	-	-	-	-	-	-	-	-	-	-	-	-	-	-	+	+
IS. 15	Sp 14	+	+	+	+	+	+	+	+	-	-	-	-	-	-	-	-	-	-	-	-	-	-	-	-	-	+	-
IS.16	SpLE 2	ND	ND	ND	ND	ND	ND	ND	ND	ND	ND	ND	ND	ND	ND	ND	ND	ND	ND	ND	ND	ND	ND	ND	ND	ND	ND	ND
IS. 17	back	+	+	-	-	-	-	-	-	-	-	-	-	-	-	-	-	-	-	-	-	-	-	-	-	-	+	+
IS. 18	Sp 15 stx1	+	+	-	-	+	-	-	-	-	-	-	-	-	-	-	-	-	-	-	-	-	-	-	-	-	+	+
IS. 19	back	+	-	+	+	+	-	-	+	-	-	-	-	-	-	-	-	-	-	-	-	-	-	-	-	-	+	-
IS. 20	Sp 17	+	-	+	+	+	-	+	+	-	-	-	-	-	-	-	-	-	-	-	-	-	-	-	-	-	+	-
IS. 21	SpLE3	+	+	-	+	+	+	+	+	-	-	-	-	-	-	-	-	-	-	-	-	-	-	-	-	-	+	+
IS.22	back	ND	ND	ND	ND	ND	ND	ND	ND	ND	ND	ND	ND	ND	ND	ND	ND	ND	ND	ND	ND	ND	ND	ND	ND	ND	ND	ND
IS. 23	SpLE 5	+	+	-	-	+	-	+	+	-	-	-	-	-	-	-	-	-	-	-	-	-	-	-	-	-	+	+
IS. 24	SpLE 1	-	+	-	-	-	-	-	-	-	-	-	-	-	-	-	-	-	-	-	-	-	-	-	-	-	-	+
IS. 25	SpLE 1	-	+	-	-	-	-	-	-	-	-	-	-	-	-	-	-	-	-	-	-	-	-	-	-	-	-	+
IS.26	933O	ND	ND	ND	ND	ND	ND	ND	ND	ND	ND	ND	ND	ND	ND	ND	ND	ND	ND	ND	ND	ND	ND	ND	ND	ND	ND	ND
IS. 27	SpLE 2	-	+	-	-	-	-	-	-	-	-	-	-	-	-	-	-	-	-	-	-	-	-	-	-	-	-	+
IS.28	933Y	ND	ND	ND	ND	ND	ND	ND	ND	ND	ND	ND	ND	ND	ND	ND	ND	ND	ND	ND	ND	ND	ND	ND	ND	ND	ND	ND
IS. 29	Sp 1	-	-	+	+	-	-	-	+	-	-	-	-	-	-	-	-	-	-	-	-	-	-	-	-	-	-	-
IS. 30	Sp 4	-	-	+	-	-	-	-	-	-	-	-	-	-	-	-	-	-	-	-	-	-	-	-	-	-	-	-
IS. 31	Phage	-	-	+	+	-	-	-	-	+	+	-	+	+	+	-	-	-	-	-	-	-	-	-	-	-	-	-
IS. 32	back	-	-	+	+	-	-	-	+	-	-	-	-	-	-	-	-	-	-	-	-	-	-	-	-	-	-	-
IS. 33	Sp 13	-	-	+	+	-	-	-	-	-	-	-	-	-	-	-	-	-	-	-	-	-	-	-	-	-	-	-
IS. 34	back	-	-	+	+	-	-	-	-	-	-	-	-	-	-	-	-	-	-	-	-	-	-	-	-	-	-	-
IS. 35	Sp 5 stx 2	-	-	+	+	-	-	-	+	-	-	-	-	-	-	-	-	-	-	-	-	-	-	-	-	-	-	-
IS.36	back	ND	ND	ND	ND	ND	ND	ND	ND	ND	ND	ND	ND	ND	ND	ND	ND	ND	ND	ND	ND	ND	ND	ND	ND	ND	ND	ND
IS. 37	Phage	-	-	+	+	-	-	-	-	-	-	-	-	-	-	-	-	-	-	-	-	-	-	-	-	-	+	-
IS. 38	back	-	-	+	+	-	-	-	-	-	-	-	-	-	-	-	-	-	-	-	-	-	-	-	-	-	-	-
IS. 39	(*gne *gene)	-	-	-	-	-	+	-	-	-	-	-	-	-	-	-	-	-	-	-	-	-	-	-	-	-	-	-
IS. 40	pO157	+	-	-	-	+	-	-	-	-	-	-	-	-	-	-	-	-	-	-	-	-	-	-	-	-	+	-
IS. 41	pO157	+	+	+	+	+	+	+	+	-	-	-	-	-	-	-	-	-	-	-	-	-	-	-	-	-	+	+
IS. 42	pO157	-	-	+	+	-	-	-	-	-	-	-	-	-	-	-	-	-	-	-	-	-	-	-	-	-	+	+
IS.43	**pO157**																											
IS. 44	pO157	-	-	+	+	-	-	-	-	-	-	-	-	-	-	-	-	-	-	-	-	-	-	-	-	-	-	-
IS. 45	pO157	-	-	-	+	-	-	-	-	-	-	-	-	-	-	-	-	-	-	-	-	-	-	-	-	-	-	-
IS. 46	back	-	-	-	+	-	-	-	-	+	+	-	-	-	-	-	-	-	-	-	-	-	-	-	-	-	-	-
IS.47	back	ND	ND	ND	ND	ND	ND	ND	ND	ND	ND	ND	ND	ND	ND	ND	ND	ND	ND	ND	ND	ND	ND	ND	ND	ND	ND	ND
IS.48	**pO157**	ND	ND	ND	ND	ND	ND	ND	ND	ND	ND	ND	ND	ND	ND	ND	ND	ND	ND	ND	ND	ND	ND	ND	ND	ND	ND	ND

IS*629 *sites were numbered from 1 - 47 (NR) starting with all sites in Sakai, followed by all additional, unshared sites from EDL933, EC4115, the sites found in the plasmids and unshared sites of strain TW1435. The newly found IS*629 *insertion in O *rough*:H7 strain MA6 was numbered IS.39 [4]. A1 - A6 are strains belonging to the different clonal complexes.

Sp - Phage; SpLE - Phage-like element; back - backbone; ND -Not determined, primers failed to amplify the region.

**Figure 3:**
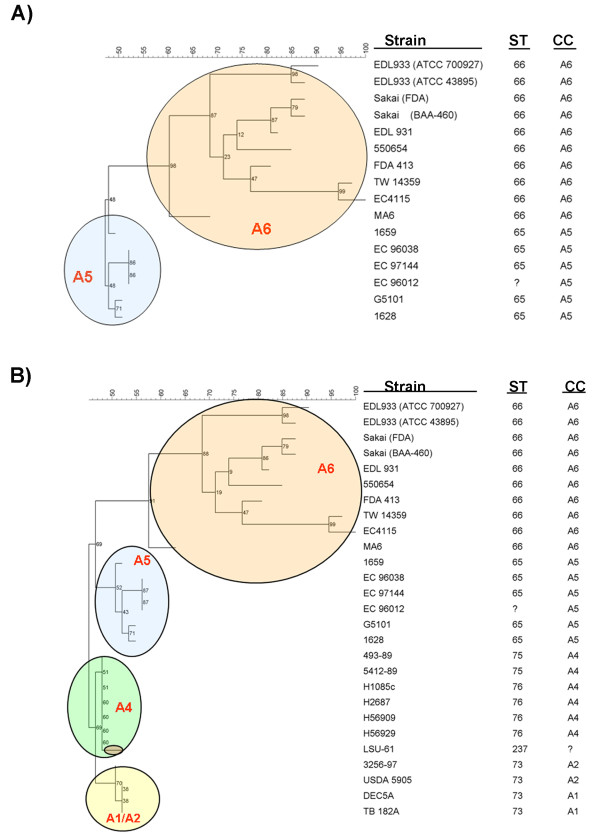
**Evolutionary significance of IS*629 *in the emergence of *E. coli *O157:H7**. A) Maximum parsimony tree obtained using the distribution of IS*629 *and IS*629 *target sites in the 14 O157:H7 strains analyzed in the present study (Table 3 and Additional file 4, Table S3). B) Maximum parsimony tree obtained using IS*629 *target sites for the 27 strains analyzed in the present study (Additional file 4, Table S3). The colored ellipses mark the different CCs. CC - clonal complex; ST - sequence type.

Figure 1B shows a maximum parsimony tree obtained for A5 and A6 CC strains using IS*629 *presence/absence in the target site and presence/absence of IS*629 *target site (chromosome or plasmid region) (Table 3 and Additional file 4, Table S3). Strains belonging to A1, A2, and A4 CCs were not included in this analysis because they either lack IS*629 *(A4) or IS*629 *is located in other regions on the chromosome than the ones determined for O157:H7 strains. The parsimony tree allowed to separate strains belonging to A5 from A6 strains as proposed in the stepwise model (Figure 1 and 3A) [10,12]. Furthermore, it showed the existence of high diversity among A5 and A6 CC strains similar to what has been shown by PFGE [11]. The validity of this analysis needs to be explored further using more O157:H7 strains belonging to either A5 or A6 CCs. Besides using 25 different strains for the analysis, we also included additional Sakai and EDL933 strains. Sakai strains were one from ATCC (BAA-460) and the other from a personal collection (FDA). EDL933 strains were provided by ATCC whereby strain EDL933 700927 derived from EDL933 43895. PFGE analysis showed only minimal changes between the original (ATCC) and the derived ones confirming their identity (data not shown). The analysis using the IS*629 *distribution also showed minimal changes in the IS*629 *distribution as well among the Sakai and EDL933 strains. The use of IS*629 *presence/absence in specific regions has been used before to help detecting outbreak related strains as described by Ooka, et al (2009) and for population genetics analysis as described by Yokoyama, et al (2011), and appears to be a promising and adequate technique to distinguish closely related O157:H7 strains. However, both methodologies takes no notice of the information about the presence of the region where IS*629 *was inserted into. The presence/absence of a specific region in *E. coli *O157:H7 chromosomes, irrelevant of the presence of IS*629*, could provide additional information regarding relatedness among those strains.

### IS*629 *insertion site prevalence in the strains belonging to the stepwise model of emergence of *E. coli *O157:H7

PCR analysis for the presence of IS*629 *insertion sites showed that sites located on the chromosomal backbone structure were present in all tested strains from the different clonal complexes (Table 4 and Additional file 4). However, neither A1, A2, nor A4 CC strains harbored any IS*629 *in backbone IS*629 *insertion sites.

**Table 4:** Presence of IS*629 *target sites on the backbone

IS*629 *target sites	A1	A2	A3	A4	A5	A6
**IS.10**	**+/-**	+	**NA**	+	+	**+/-**
**IS.11**	+	+	**NA**	+	+	+
**IS.13**	+	+	**NA**	+	+	+
**IS.17**	+	+	**NA**	+	+	+
**IS.19**	+	+	**NA**	+	+	+
**IS.32**	+	+	**NA**	+	+	+
**IS.34**	+	+	**NA**	+	+	+
**IS.38**	+	+	**NA**	+	+	+
**IS.39**	+	+	**NA**	+	+	+
**IS.46**	-	-	**NA**	**+/-**	+	+

NA, not applicable; + presence; - absence; +/- present in some strains.

Contrary to what was observed in the well-conserved backbone, IS*629 *insertion sites in prophages and prophage-like elements in different strains were found to be highly variable (Table 5 and Additional file 4, Table S3). As seen for the backbone IS*629 *insertion sites, some of the phage associated IS*629 *insertions sites were present in A1, A2 and A4 CC strains; however they lacked IS*629*. Many of the IS*629 *sites on phages were unique to the A6 CC strains (7 of 13) suggesting that they are strain-specific. This result underscores significant differences in the presence of phage-related sequences between the strains belonging to the stepwise model of *E. coli *O157:H7.

**Table 5:** Presence of phage or phage-like associated IS*629 *target sites

IS*629 *target sites	A1	A2	A3	A4	A5	A6
**Sp 1**	_	_	**NA**	_	_	+
**Sp 2**	+	+	**NA**	+	+	+
**Sp 4**	+	+	**NA**	+	+	+
**Sp 5**	_	_	**NA**	_	_	+
**Sp 8**	_	_	**NA**	_	_	+
**Sp 12**	_	+	**NA**	+	+	+
**Sp 13**	_	_	**NA**	_	_	+
**Sp 14**	_	_	**NA**	+	+	+
**Sp 17**	_	_	**NA**	_	_	+
**SpLE 1**	_	_	**NA**	_	+	+
**SpLE 2**	_	_	**NA**	_	_	+
**SpLE 3**	_	_	**NA**	_	_	+
**SpLE 5**	_	_	**NA**	_	+	+

Sp - Phage; SpLE - Phage-like element; NA - not applicable; + presence; - absence.

The two IS*629 *insertions in O55 and its corresponding plasmid pO55 were observed to be present in only one ancestral A2 and both A1 CC strains (data not shown). A6, A5, and A4 CC strains as well as A2 CC strain 3256-97 (IS*629*-deficient) lacked the IS*629 *insertion site in these regions. Interestingly, strain LSU-61 which carries multiple characteristics for O157:H7 and is thought to be ancestral to A5 CC strains (Feng et al 2007), appeared to carry the truncated genomic IS*629 *insertion.

Since the strains belonging to the stepwise model share variable IS*629 *insertion sites we reconstructed their evolutionary path using this information. A parsimony tree using the IS*629 *target sites presence/absence produced a tree that was nearly analogous to the proposed model of stepwise evolution for O157:H7 from ancestral O55:H7 strains [10], with A1/A2 CC strains at the base of the tree, followed by A4 CC, A5 CC and A6 CC strains in that order (Figure 3B).

### Phylogenetic analysis of IS*629 *elements in the four *E. coli *O157:H7 and O55:H7 genomes

**Figure 4:**
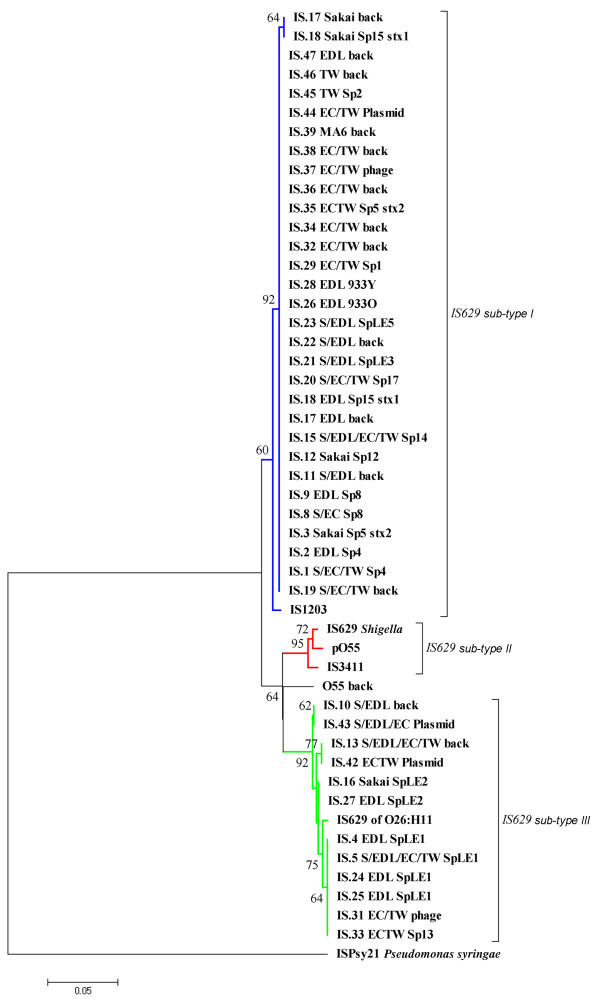
**Phylogenetic tree of IS*629 *in *E. coli *O157:H7 and O55:H7 showing the three different IS*629 *sub-types present on those five genomes**. IS*629 *sub-type I differed from sub-type II by 4% (> 55 bp) and sub-type II differed from sub-type III by 5% (> 60 bp). IS*629 *sub-type II was only present in O55:H7 genome (A1/A2 CC) while sub-type I and III were present in all O157:H7 genomes (A6 CC). The evolutionary history was inferred using the Minimum Evolution method [31]. The tree is drawn to scale, with branch lengths in the same units as those of the evolutionary distances used to infer the phylogenetic tree. Bootstrap support when above 50% is shown at nodes. Sp- prophages; SpLE - prophage-like elements; and back - backbone.

The phylogenetic analysis of IS*629 *elements revealed that IS*629 *in *E coli *O157:H7 can be divided into three different sub-types (Figure 4). That is, IS*629 *of sub-type I and II differ in average 4% (> 55 bp) while sub-type II and III differed by 5% (> 60 bp). Sub-type I appears to be most closely related to those of IS*1203 *(IS*629 *isoform) found in O111:H- [18]. IS*629 *sub-type II appears to be most closely related to those of IS*629 *found in *Shigella *[19]. IS*629 *sub-type III appears to be most closely related to those of IS*629 *found in *E. coli *O26:H11 [20]. Therefore, analysis of all targeted IS*629 *elements showed that strains from A6 CC seem to carry both IS*1203 *(sub-type I) and IS*629 *(sub-type III) whereby the ancestral O55:H7 strain carries IS*629 *(sub-type II). Since IS*629 *sub-type II found in the ancestral O55:H7 strain is significantly different from the other two IS*629 *sub-types (O157:H7 strains) and sub-type II is no longer present in certain O157:H7 strains (A6 CC), these data imply that IS*629 *sub-type I and III were recently acquired by *E. coli *O157:H7 strains after the separation from the sub-lineage leading to the A4 CC strains therefore not carrying IS*629*.

## Discussion

IS elements are in general regarded as genetic factors that significantly contribute to genomic diversification and evolution [7]. It was determined by Ooka et al (2009) that IS elements IS*629 *and IS*Ec8*, found in the O157:H7 lineage, serve as an important driving force behind the genomic diversity. However, only a few genome-wide studies have been conducted to compare IS distributions in closely related genomes. In our study we determined that IS*629 *insertions in *E. coli *O157:H7 are widespread distributed on the genome and differ significantly from strain to strain. Although the ancestral O55:H7 strain carried only two IS*629 *with one on the chromosome and one on the pO55 plasmid, the four O157:H7 genomes carried between 22 and 25 IS*629 *copies on the chromosome and the corresponding pO157 plasmid.

IS*629 *does not seem to specifically integrate in sequence-based target sites, which explains the highly diverged flanking sites found in the genomes we examined. Sequence-specific insertion is exhibited to some degree by several elements and varies considerably in stringency [21]. Other elements exhibit regional preferences which are less obvious to determine [21]. IS elements frequently generate short target site duplication (TSD) flanking the IS upon insertion [21]--this feature was also observed for IS*629 *in the four O157:H7 strains. IS*629 *duplicated between 3 to 4 base pairs at the insertion site and was observed for 21 of the 47 IS*629 *insertion sites with matching identical base pairs up- and down-stream of IS*629*. A comparison of 21 TSDs created by IS*629 *in the four strains analyzed here did not reveal as many similarities as observed previously by Ooka et al (2009). The comparison of 25 bp up- and downstream of each insertion site did not show any similarities or patterns which would have suggested a target preference or "hot-spot" for IS*629 *insertions. Hence, insertion site specificity for IS*629 *remains unknown. However, IS*629 *is frequently surrounded by other IS elements ('IS islands') and was found in the same gene (*gne*) inserted in different sites [4,13]. Although no specific "hot-spot" for IS629 insertions was observed, it seems highly possible that mobile elements like plasmids, phages or phage-like elements could have functioned as vectors for IS629 introduction into O157:H7 genomes. These observations suggest that an insertion might occur preferentially in a region of the chromosome however these events may not be sequence specific.

IS*629 *insertion sites located on the backbone seem to be conserved in almost all of the strains studied here, whereby sites located on phages and phage-like areas appear to differ between all strains. These findings affirm the presence of regions of genomic stability and regions of genomic variability that exist within O157:H7 populations and closely related strains. It is noteworthy that sites associated with phages seem to be present predominantly in closely related strains. The majority of the phages present in the A6 CC strains appear to be unique to this complex. Since bacteriophages are known to contribute to the diversification of bacteria [22], they seem to be a major determinant in generating diversity among O55:H7, O157:H- and O157:H7 strains. The comparison of IS*629 *prevalence in A5 and A6 CC as well as IS*629 *insertion site prevalence in all strains allowed distinguishing strains from different complexes as it has been proposed in the evolution model for O157:H7 (Figure 1A) [11]. Adding the "same" strain from different collections, Sakai and EDL933 allowed confirmation of the stability of IS*629 *sites. Minimal changes in IS*629 *presence/absence were observed and could have occurred due to different storage conditions and passages. Despite these subtle changes, strains grouped tightly together on the parsimony tree. Therefore, this analysis can be used to further distinguish closely related O157:H7 strains. These findings are in agreement with a recently described IS*629 *analysis in three O157 lineages [23]. Similarly to what was determined for A6 and A5 CC strains, Yokoyama et al (2011) determined that IS*629 *distribution was biased in different O157 lineages, indicating the potential effectiveness of IS-printing for population genetics analysis of O157. Furthermore, Ooka et al. (2009) found that IS-printing can resolved about the same degree of diversity as PFGE. Since A1, A2 and A4 CC strains did not share IS*629 *insertions, their population genetics analysis however, remains limited to closely related O157:H7 strains.

Comparison of IS*629*s found in O157:H7 and O55 pointed out extensive divergence between these elements. At least three different IS*629 *types could be distinguished differing in 55 to 60 bp. The O157:H7 strains carry IS*629 *elements subtype I and III whereby O55:H7 carries type II only. It is notable that only four nucleotide differences were observed among seven housekeeping genes comprising a current MLST scheme http://www.shigatox.net/ecmlst/cgi-bin/dcs between A1 CC strain DEC5A and A6 CC strain Sakai. These two strains, in particular, are taken to represent the most ancestral and most derived *E. coli*, respectively, in the stepwise evolutionary model for this pathogen. If the IS*629 *type I and III observed in A6 CC strains resulted from divergent evolution of IS*629 *type II, the amount of changes observed among these IS types should be similar to those observed for the MLST loci examined above. However, the number of nucleotide substitutions between IS*629 *type I and III in O157:H7 from type II in O55:H7 was 10-fold higher. Thus, the differences between IS*629 *types are more significant than those observed for housekeeping genes. This indicates that IS*629*-type II was most likely lost and IS*629*-type I and III were acquired independently in distinct *E. coli *O157:H7 lineages. Further supporting this thesis was the fact that one of the IS*629 *type II copies was found on the pO55 plasmid, which was subsequently lost during evolution towards O157:H7 strains. The other IS*629 *copy in O55, with a unique internal deletion, is located in the chromosome and appears to be part of a mobile region [24] which is absent in O157:H7 strains.

Interestingly, the ancestral IS*629*-deficient A2 O55:H7 strain 3256-97 is also lacking both IS*629 *associated regions found in the O55:H7 strains. Our analysis of common IS*629 *target sites demonstrated that strain 3256-97 seems to be more closely related to A4 and A5 CC strains than other A1 and A2 strains. Therefore, it is likely that IS*629 *has been lost in strain 3256-97 as well as in the hypothetical A3 precursor. These results may indicate that strain 3256-97 or a similar strain lacking IS*629 *might have given rise to IS*629*-deficient A4 CC strains.

*E. coli *O157:H7 strains carry multiple IS*629 *copies while the non-pathogenic K-12 strain lacks IS*629 *but carries other IS elements. Other pathogenic *E. coli *strains, amongst the top six non-O157 STEC O26:H11, O111:H- and O103:H2 [25], also harbor various copies of IS*629 *elements in their genomes. Genome sequences for the other three most important pathogenic non-O157 STEC; O45, O145, and O121 are not available to date thus the presence of IS*629 *elements is unknown. Interestingly, they also share the same reservoir with O157:H7 (*e.g*. cattle), shiga-toxins, haemolysin gene cluster, other virulence factors and several phages and phage-like elements [25]. Ooka et al (2009) postulated that IS-related genomic rearrangements may have significantly altered virulence and other phenotypes in O157 strains. These findings suggest that IS*629 *might not only have a great impact in their genomic evolution but might increase the pathogenicity of those strains as well.

## Conclusions

The genomic sequence analysis showed that IS*629 *insertion sites exhibited a highly biased distribution. IS*629 *was much more frequently located on phages or prophage-like elements than in the well-conserved backbone structure, which is consistent with the observations by Ooka et al (2009). IS*629 *was found to be present in the A1 and one of two A2 CC strains examined as well as in all the O157:H7 strains of A5 and A6 CC, however it was totally absent in the 6 examined SFO157 strains of A4 CC. The A4 CC strains are related to but on a divergent evolution pathway from O157:H7. These results suggest that the absence of IS*629 *in A4 strains probably occurred during the divergence, but it is uncertain if it contributed to the divergence. Overall, IS*629 *had great impact on the genomic diversification of the *E. coli *O157:H7 lineage and might have contributed in the emergence of the highly pathogenic O157:H7.

## Methods

### Bacterial strains

The bacterial strains used in this study are listed in Table 2 and were chosen to represent typical EHEC and EPEC strains from the different clonal complexes from the evolution model for *E. coli *O157:H7 [11] with different serotypes (O157:H7, O157:H- and O55:H7) and different characteristics (e.g. β-glucuronidase activity (GUD), sorbitol fermentation (SOR).

### "In silico" analysis

Various *E. coli *O157:H7 and non-O157 chromosomes and pO157 plasmids (Additional file 2, Table S1) deposited at the National Center for Biotechnology Information (NCBI) database were queried for IS*629 *(accession number X51586) presence and insertion loci using BLAST analysis. Furthermore, approximately 400 bp up- and downstream of the flanking regions of each new localized IS*629 *in the chromosome and the plasmids were compared with each other. We investigated whether an IS*629 *was also present in the other strains or appears exclusively in either the chromosome or the plasmids.

### Nucleic acid extraction and determination of IS*629 *presence

DNA used as the template for PCR was prepared from overnight cultures grown in Luria-Bertani Broth (LB) and purified using the MASTER PURE™ DNA Purification kit (EpiCentre, Madison, WI). For determining IS*629 *presence in the *E. coli *strains, we conducted a "touchdown" multiplex PCR using IS*629*-specific primers targeting conserved regions of the insertion element previously described by Ooka et al. (2009): IS*629*-insideF (5'- GAACGTCAGCGTCTGAAAGAGC-3') and IS*629*-insideR (5'- GTACTCCCTGTTGATGCCAG-3') and specific 16S rDNA primers: SRM86 (5'- AGAAGCACCGGCTAACTC -3') [7] and SRM87 (5'- CGCATTTCACCGCTACAC-3') [26]. The latter were used as internal amplification control. PCR amplifications were performed using 0.5 ng of template DNA and in a final volume of 30 μl. The PCR reaction mixture contained 2.5 U of HotStart Taq Polymerase (Qiagen, Valencia, CA), 1X Taq polymerase buffer, 2.0-3.5 mM MgCl_2_, 400 μM each deoxynucleoside triphosphate (dNTP), 300 nM each IS*629 *primer pair, and 300 nM each 16S rDNA primer pair. The "touchdown" PCR [27] conditions were: 1 cycle of 95°C for 15 min; 10 cycles of 95°C for 30 s, 69-59°C (-1°C/cycle) for 15 s and 72°C for 1:30 min; followed by 35 cycles consisting of 95°C for 30 s, 58°C for 20 s, and 72°C for 1.5 min, and a final extension at 72°C for 4 min. Amplicons were visualized on a 1% agarose gel in Tris-Borate EDTA (TBE) buffer containing 0.3 μg/ml ethidium bromide.

### Determination of IS*629 *specific location and IS*629 *insertion sites

For the analysis of the IS*629 *insertion sites, primers were designed to target the different IS*629 *flanking regions in each strain and the plasmids. The presence/absence of amplicons would determine the presence/absence of the specific insertion sites and the sizes of each amplicons would indicate the presence/absence of IS*629 *at those loci. Potential primers were analyzed for their ability to produce stable base pairing with the template using the NetPrimer software (PREMIER Biosoft International http://www.premierbiosoft.com/netprimer/netprlaunch/netprlaunch.html). The size of the PCR products were between 1,500 - 2,500 bp in the case of IS*629 *presence in a strain or between 200 - 800 bp in the case that the specific flanking region existed in the chromosome but did not contain an IS*629 *element. Each multiplex PCR contained a set of 16S rDNA primers as PCR internal control (either set SRM86/SRM87 or VMP5 (5'-AGAAGCACCGGCTAACTC-3') and VMP6 (5'-CGCATTTCACCGCTACAC-3') [28]), and IS*629 *insertion site specific primers. The list of the 40 primer combinations for each IS*629 *site and PCR conditions can be found in Additional file 5, Table S4.

### IS*629 *presence/absence parsimony tree analysis

IS*629 *PCR fragments sizes indicating IS*629 *presence/absence and IS*629 *target site presence/absence identified by PCR using primers specific for each IS*629 *observed in 4 *E. coli *O157:H7 genomes were entered as binary characters (+ or -) into BioNumerics version 6.0 (Applied Maths, Saint-Martens-Latem, Belgium). IS*629 *presence/absence and IS*629 *target site presence/absence were used to create a phylogenetic parsimony tree rooted to A5 CC strains for A5/A6 CC strains analysis (Figure 1B) and statistical support of the nodes was assessed by 1000 bootstrap re-sampling. IS*629 *target site presence/absence were used to create a phylogenetic parsimony tree rooted to A1/A2 CC strains for strains of the entire model (A1 - A6) (Figure 1C) and statistical support of the nodes was assessed by 1000 bootstrap re-sampling.

### IS*629 *phylogenetic analysis

Minimum evolution tree for IS*629 *sequences present in 4 *E. coli *O157:H7 genomes, two IS*629 *in O55:H7 genome, IS*629 *sequences from *Shigella*, two other IS*629 *isoforms (IS*1203 *and IS*3411*), and IS*Psy21 *(a member of the IS*3 *family and sharing only 68% homology with IS*629*) as out-group (*Pseudomonas syringae *pv. savastanoi TK2009-5) was constructed using Mega version 4.0 [29]. The evolutionary distances were computed using the Kimura 2-parameter method [30] and are in the units of the number of base substitutions per site. All positions containing gaps and missing data were eliminated from the dataset (Complete deletion option). There were a total of 299 positions in the final dataset. The statistical support of the nodes in the ME tree was assessed by 1000 bootstrap re-sampling.

## Authors' contributions

LVR conceived the study, participated in the experimental design, performed all the experiments, and participated in the production of the draft of the manuscript. MF participated in the experimental design, and production of the draft of the manuscript. NGE participated in the experimental design and coordination, performed most of the sequence analysis and phylogeny, and participated in production of the draft of the manuscript. All authors have read and approved the final manuscript.

## Supplementary Material

Additional file 1 **"Figure S1"**. Schematic representation of the strategy used for primer design. Primer pairs: A: presence/absence of IS*629 *at specific loci, B: IS*629 *internal primer. A) Amplification product for locations where the IS629 element is present; B) Amplification product for locations where the IS629 element is absent, although the up-and downstream flanking region is present in the genome but not carrying an insertion. Click here for file

Additional file 2 **"Table S1"**. Genomes and plasmids investigated by "in silico" analysis. Click here for file

Additional file 3 **"Table S2"**. IS*629 *insertion sites in O157:H7 strains with complete genomes available in Genbank (Additional Table 1). In bold are the locations shared by the four O157:H7 strains. The direct repeats (duplication are in red). IS*629 *sites were numbered from 1 - 47 starting with all sites in Sakai, followed by all additional, unshared sites from EDL933, EC4115, the sites found in the plasmids and unshared sites of strain TW1435. The newly found IS*629 *insertion in O *rough*:H7 strain MA6 was numbered IS.39. Click here for file

Additional file 4 **"Table S3"**. IS*629 *target site presence/absence in CC strains from the O157:H7 stepwise evolutionary model. Click here for file

Additional file 5 **"Table S4"**. Primer sequences for the amplification of each flanking IS*629 *regions on the four *E. coli *genomes available (see Additional Table 2). If IS absent size equal to 0 bp means that the primer pair was designed with one target region inside IS*629 *therefore the IS*629 *target site could not be observed. Click here for file
