# Supplementary material for: Prevalence, distribution and evolutionary significance of the IS629 insertion element in the stepwise emergence of Escherichia coli O157:H7
Source: BMC Microbiol. 2011 Jun 14;11:133. doi: 10.1186/1471-2180-11-133 (PMC3271280; doi:10.1186/1471-2180-11-133)
Supplement: Additional file 1 — "Figure S1". Schematic representation of the strategy used for primer design. Primer pairs: A: presence/absence of IS629 at specific loci, B: IS629 internal primer. A) Amplification product for locations where the IS629 element is present; B) Amplification product for locations where the IS629 element is absent, although the up-and downstream flanking region is present in the genome but not carrying an insertion. [file 1471-2180-11-133-S1.docx]

Supplementary Table 1 - Genomes and plasmids investigated by “in silico” analysis.

| **Strain** | **Serotype** | **Accession No.** | |
| --- | --- | --- | --- |
|  |  | **Genomes** | **Plasmids** |
| Sakai | O157:H7 | NC_002695 | AB011549 (pO157) |
| EDL933 | O157:H7 | AE005174 | AF074613 (pO157) |
| EC4115 | O157:H7 | NC_011353 | CP001163 (pO157) |
| TW14359 | O157:H7 | CP001368 | CP001369 (pO157) |
| 3072/96 | SFO157 | NA | NC_009602.1 (pSFO157) |
| CB9615 | O55:H7 | NC_013941 | CP001847.1 (pO55) |

NA - not available.
