# Supplementary material for: Prevalence, distribution and evolutionary significance of the IS629 insertion element in the stepwise emergence of Escherichia coli O157:H7
Source: BMC Microbiol. 2011 Jun 14;11:133. doi: 10.1186/1471-2180-11-133 (PMC3271280; doi:10.1186/1471-2180-11-133)
Supplement: Additional file 2 — "Table S1". Genomes and plasmids investigated by "in silico" analysis. [file 1471-2180-11-133-S2.DOCX]

**Supplementary Table 2** - IS*629* insertion sites in O157:H7 strains with complete genomes available in Genbank (Supplementary Table 1). In bold are the locations shared by the four O157:H7 strains. The direct repeats (duplication are in red). IS*629* sites were numbered from 1 - 47 starting with all sites in Sakai, followed by all additional, unshared sites from EDL933, EC4115, the sites found in the plasmids and unshared sites of strain TW1435. The newly found IS*629* insertion in O *rough*:H7 strain MA6 was numbered IS.39 [1].

| **NR.** | **Phage or backbone** | **Upstream** | **Downsteam** | **SAKAI** | | **EDL933** | | | **EC4115** | | | TW14359 | | | |
| --- | --- | --- | --- | --- | --- | --- | --- | --- | --- | --- | --- | --- | --- | --- | --- |
|  |  | **sequence** | | **location** | | **location** | | | **location** | | | **location** | | | |
| IS.1 | Sp 4 | ATATGCGGAT | TTAATTAATG | 1182129 | 1183201 |  |  | |  |  | | 1187070 | 1188379 | |  |
| IS.2 | Sp 4 | ATATGCGGAT | TTAATTAATG |  |  |  |  | | 1185471 | 1186780 | |  |  | |  |
| IS.3 | Sp 5 stx2 | AATGCGAAAG | AAAG**T**GGCGA | 1270361 | 1271670 |  |  | |  |  | |  |  | |  |
| IS.4 | SpLE 1 | AGGAGGCTGG | TGGTGATTGT |  |  | 1120064 | 1121373 | |  |  | |  |  | |  |
| IS.5 | SpLE 1 | AGGAGGCTGG | TGGTGATTGT | **1431898** | **1433207** | **1515671** | **1516980** | | **1375483** | **1376792** | | **1375770** | **1377079** | |  |
| IS.6 | SpLE 1 | GAATCAGGTT | truncated |  |  | 1129492 | 1129750 | |  |  | |  |  | |  |
| IS.7 | SpLE 1 | GAATCAGGTT | truncated | **1441326** | **1441584** | **1525098** | **1525356** | | **1384911** | **1385169** | | **1385198** | **1385456** | |  |
| IS.8 | Sp 8 | TGTTGATAAA | CCACTGATGC | 1663333 | 1664642 |  |  | | 1600004 | 1601313 | |  |  | |  |
| IS.9 | Sp 8 | CTGGTCTTTG | CCACTGATGC |  |  | 1754739 | 1756048 | |  |  | |  |  | |  |
| IS.10 | backbone | TCTTGCCATC | ATCAATCACT | 1685465 | 1686774 | 1776871 | 1778180 | |  |  | |  |  | |  |
| IS.11 | backbone | CGCTGACTTT | TTTCGGTCAA | 1909167 | 1910476 | 2176690 | 2177999 | |  |  | |  |  | |  |
| IS.12 | Sp 12 | ACGATGGCCT | CCTTTCAGTT | 2205544 | 2204235 |  |  | |  |  | |  |  | |  |
| IS.13 | backbone | AAAAACCCTG | CTGTTAATAA | **2454183** | **2455492** | **2529386** | **2530695** | | **2360477** | **2361786** | | **2359458** | **2360767** | |  |
| IS.14 | Sp 13 | truncated | ATCTGCTGGC | **2603612** | **2604668** | **2678823** | **2679879** | | **2509906** | **2510962** | | **2508887** | **2509943** | |  |
| IS.15 | Sp 14 | TTAGCCAGTA | GTACTTCTCC | **2693477** | **2694786** | **2141966** | **2143277** | | **2601091** | **2602400** | | **2600071** | **2601380** | |  |
| IS.16 | SpLE 2 | AAGAAGGTGG | GTCTCAGTCC | 2741821 | 2743130 |  |  | |  |  | |  |  | |  |
| IS.17 | backbone | CAACAAGGAT | GATGAAATAG | 2890776 | 2892085 | 2961007 | 2962316 | |  |  | |  |  | |  |
| IS.18 | Sp 15 stx1 | CTCCTTGCCC | CCCATTTCAT | 2912202 | 2913511 | 2761491 | 2762801 | |  |  | |  |  | |  |
| IS.19 | backbone | CCAGCCGGGC | GGATGCCCCA | 3088838 | 3090147 |  |  | | 3130200 | 3131509 | | 3085071 | 3086380 | |  |
| IS.20 | Sp 17 | GTGCGCCATC | GTTCGGCGGT | 3480689 | 3481998 |  |  | | 3584797 | 3586106 | | 3539674 | 3540983 | |  |
| IS.21 | SpLE3 | ACAAACTGAT | CACAGCCAGA | 3869333 | 3870642 | 3936646 | 3937955 | |  |  | | 3928456 | 3929765 | |  |
| IS.22 | backbone | ATTAAAACCA | GGGCGATGTT | 4033206 | 4034515 | 4100422 | 4101731 | |  |  | |  |  | |  |
| IS.23 | SpLE 5 | ATTAGCTCAG | CAGAATTGAT | 5349269 | 5350578 | 5379272 | 5380581 | |  |  | |  |  | |  |
| IS.24 | SpLE 1 | AAATACGCGC | CGCTGGTGCG |  |  | 1142937 | 1144246 | |  |  | |  |  | |  |
| IS.25 | SpLE 1 | AAATACGCGC | CGCTGGTGCG |  |  | 1538543 | 1539852 | |  |  | |  |  | |  |
| IS.26 | 933O | ATATGCGGAT | CCCATTTCAT |  |  | 1896355 | 1897664 | |  |  | |  |  | |  |
| IS.27 | SpLE 2 | AAGAAGGTGG | GTTTTCACTT |  |  | 2818186 | 2819495 | |  |  | |  |  | |  |
| IS.28 | 933Y | GATTACCCTG | GTACTTCTCC |  |  | 3550226 | 3551537 | |  |  | |  |  | |  |
| IS.29 | Sp 1 | CAATTGGTTA | TTATGGCTGT |  |  |  |  | | 304028 | 305337 | | 304028 | 305337 | |  |
| IS.30 | Sp 4 | TGACGTGGTG | ATGACACACC |  |  |  |  | | 1189927 | 1191236 | |  |  | |  |
| IS.31 | Phage | GAAATGGACT | GACTTCATGA |  |  |  | |  | 1321708 | | 1320399 | 1321994 | 1320685 |  |  |
| IS.32 | backbone | TACCAGAAAC | AACTGATGCA |  |  |  |  | | 2157429 | 2158738 | | 2156410 | 2157719 | |  |
| IS.33 | Sp 13 | GCTCAGACAG | CAGTGTCAGG |  |  |  |  | | 2519243 | 2520552 | | 2519533 | 2518224 | |  |
| IS.34 | backbone | ATCAGTAGAT | GATATATTCT |  |  |  |  | | 2646308 | 2647617 | | 2645289 | 2646598 | |  |
| IS.35 | Sp 5 stx 2 | CAGTACGAGA | AGAACGACGC |  |  |  |  | | 2713108 | 2714417 | | 2712089 | 2713398 | |  |
| IS.36 | backbone | CTACGCGCAT | CATTCCTGCG |  |  |  |  | | 2971677 | 2972986 | | 2926548 | 2927857 | |  |
| IS.37 | Phage | ACGGGACTGG | TGGAGATAGT |  |  |  |  | | 3258306 | 3259615 | | 3213177 | 3214486 | |  |
| IS.38 | backbone | CGCTATAGCC | GCCAGCATCT |  |  |  |  | | 4259157 | 4260466 | | 4215198 | 4216507 | |  |
| **IS.39** | backbone  (*gne* gene) | ACATAAGCCA | CCATGGACTT |  |  |  |  | |  |  | |  |  | |  |
| IS.40 | pO157 | TACGGTACTG | GTGACAATTT | **30694** | **32003** | **11103** | **10072** | | **64171** | **63140** | | **79197** | **80230** | |  |
| IS.41 | pO157 | truncated | GTGTTTGTTA | 80618 | 79587 |  |  | |  |  | |  |  | |  |
| IS.42 | pO157 | ATGCAGTTTA | TTAAGGTTTA |  |  |  |  | | 13013 | 14322 | | 29095 | 30404 | |  |
| IS.43 | pO157 | ATCCGGTGAT | truncated | **66033** | **66338** | **88594** | **88899** | | **49585** | **49890** | | **65642** | **65947** | |  |
| IS.44 | pO157 | GTGAAGTGTG | GTGACAATTT |  |  |  |  | | 16109 | 17418 | | 32189 | 33500 | |  |
| IS.45 | pO157 | TGCTCCGAAG | AAGTGTTTCG |  |  |  |  | |  |  | | 315206 | 316515 | |  |
| IS.46 | backbone | TGCCACTGTG | GTGGATATGT |  |  |  |  | |  |  | | 2869454 | 2870763 | |  |
| IS.47 | backbone | GATTACCCTG | GTTCGGCGGT |  |  | 1871035 | 1872344 | |  |  | |  |  | |  |
| IS.48 | pO157 | TACGGTACTG | CCACTCAGTT |  |  | 53269 | 54578 | |  |  | |  |  | |  |

NR – number of IS629, starting with all sites in Sakai, then all additional, unshared sites from EDL933, followed by additional unshared sites found in EC4115 and sites found in the plasmids and unshared IS629 insertion sites of strain TW1435. Truncated – partial IS629, with deletion in various regions of the IS element. IS.39 locus is the IS*629* located inside *gne* gene in MA6 strain [1].

**References**

1. Rump LV, Feng PC, Fischer M, Monday SR: **Genetic analysis for the lack of expression of the O157 antigen in an O Rough:H7 Escherichia coli strain**. *Appl Environ Microbiol* 2010, **76:**945-947.
