# Supplementary material for: Prevalence, distribution and evolutionary significance of the IS629 insertion element in the stepwise emergence of Escherichia coli O157:H7
Source: BMC Microbiol. 2011 Jun 14;11:133. doi: 10.1186/1471-2180-11-133 (PMC3271280; doi:10.1186/1471-2180-11-133)

**Supplementary Figures**

**Supplementary Figure 1**. Schematic representation of the strategy used for primer design. Primer pairs: A: presence/absence of IS*629* at specific loci, B: IS*629* internal primer. A) Amplification product for locations where the IS629 element is present; B) Amplification product for locations where the IS629 element is absent, although the up-and downstream flanking region is present in the genome but not carrying an insertion.


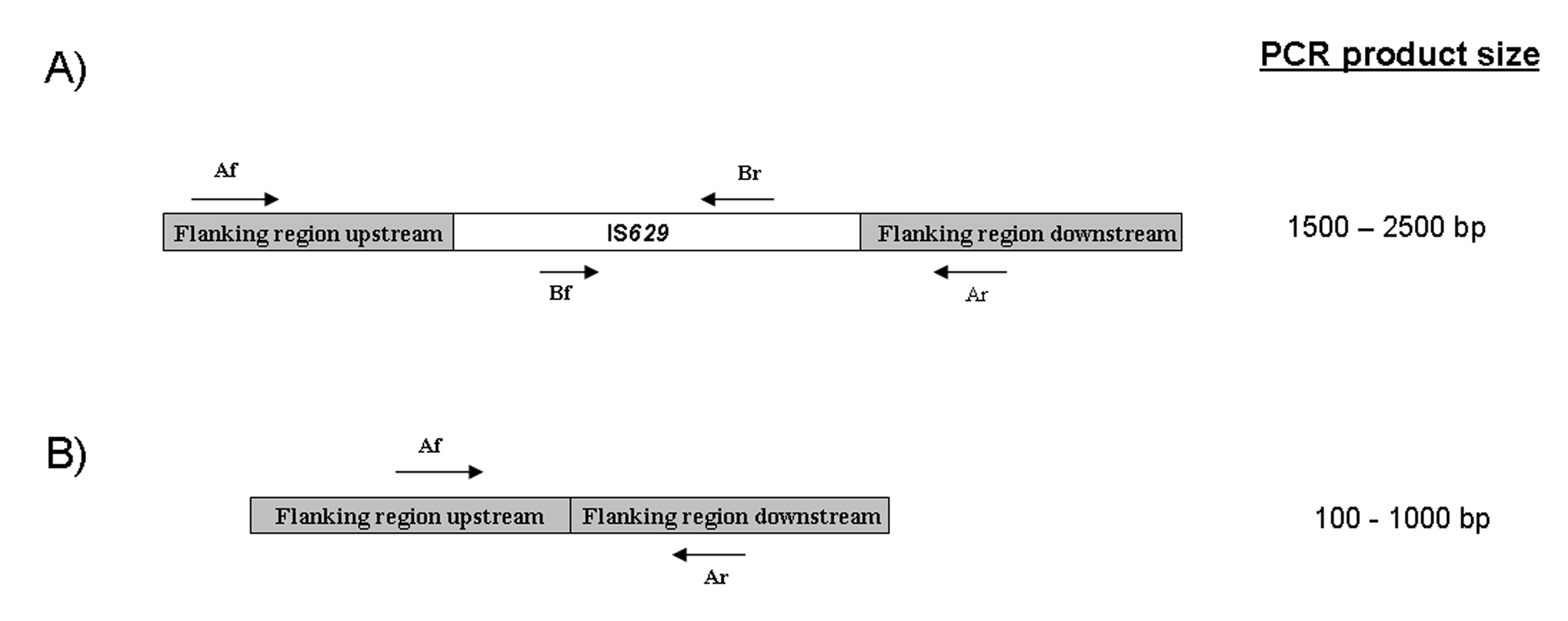

Supplement: Additional file 3 — "Table S2". IS629 insertion sites in O157:H7 strains with complete genomes available in Genbank (Additional Table 1). In bold are the locations shared by the four O157:H7 strains. The direct repeats (duplication are in red). IS629 sites were numbered from 1 - 47 starting with all sites in Sakai, followed by all additional, unshared sites from EDL933, EC4115, the sites found in the plasmids and unshared sites of strain TW1435. The newly found IS629 insertion in O rough:H7 strain MA6 was numbered IS.39. [file 1471-2180-11-133-S3.DOC]
