# Supplementary material for: Prevalence, distribution and evolutionary significance of the IS629 insertion element in the stepwise emergence of Escherichia coli O157:H7
Source: BMC Microbiol. 2011 Jun 14;11:133. doi: 10.1186/1471-2180-11-133 (PMC3271280; doi:10.1186/1471-2180-11-133)
Supplement: Additional file 5 — "Table S4". Primer sequences for the amplification of each flanking IS629 regions on the four E. coli genomes available (see Additional Table 2). If IS absent size equal to 0 bp means that the primer pair was designed with one target region inside IS629 therefore the IS629 target site could not be observed. [file 1471-2180-11-133-S5.DOCX]

**Supplementary Table 4** - Primer sequences for the amplification of each flanking IS*629* regions on the four *E. coli* genomes available (see Supplementary Table 2). If IS absent size equal to 0 bp means that the primer pair was designed with one target region inside IS*629* therefore the IS*629* target site could not be observed.

| Target | Primer | Sequences (5’ - 3’) | MgCl_2_  HotTaq | 16S rDNA  Primers | Product length [bp] | |
| --- | --- | --- | --- | --- | --- | --- |
|  |  |  |  |  | IS present | IS absent |
| IS.1 | primers that yielded specific amplicons could not be designed | | | | | |
| IS.2 | primers that yielded specific amplicons could not be designed | | | | | |
| IS.3 | Sak 3 IS FW | CCACAATGCGAAAGTGAACC | 1.5 mM | VMP5 & VMP6  0.625 μM | 1,724 | 0 |
|  | Sak 3 REV | CCATCCTGACCATCAATAACC |  |  |  |  |
| IS.4 | Sak 4 FW | CTGGTTTGTTACTCAGCATTCT | 1.5 mM | VMP5 & VMP6  0.625 μM | 1,730 | 339 |
|  | Sak 4 A REV | CAGTGACAGCAAGGACGGA |  |  |  |  |
| IS.5 | Sak 4 FW | Same as IS.4 |  |  |  |  |
|  | Sak 4 A REV |  |  |  |  |  |
| IS.6 | Sak 5 FW | TCACCATCAACGAAAACCG | 1.5 mM | VMP5 & VMP6  0.625 μM | 585 | 0 |
|  | Sak 5 REV | ATGCCAGTTCCACCATACG |  |  |  |  |
| IS.7 | Sak 5 FW | Same as IS.6 |  |  |  |  |
|  | Sak 5 REV |  |  |  |  |  |
| IS.8 | EDL 8/Sak 6 FW | ATCAGCAAAGTTGAACGAGG | 2.5 mM | 86 & 87  0.420 μM | 2027 | 717 |
|  | Sak 6 REV | GATCAAAACCGATATGTGAAT |  |  |  |  |
| IS.9 | EDL 8/Sak 6 FW | Same as IS.8 |  |  | 1638 | 328 |
|  | Sak 6 REV |  |  |  |  |  |
| IS.10 | Sak 7 FW | ATCTACAAACTTGAGCAGGCAC | 1.5 mM | VMP5 & VMP6  0.625 μM | 1,744 | 434 |
|  | Sak 7 REV | CCGATATTTCCGCATCTCC |  |  |  |  |
| IS.11 | Sak 8 FW | CCGCTAAGCCCTGAACTTT | 1.5 mM | VMP5 & VMP6  0.625 μM | 1,778 | 454 |
|  | Sak 8 REV | CCTGATAACCGACAAAATCATC |  |  |  |  |
| IS.12 | Sak 9 FW | CCAAAATCAACAAAATGCCA | 3.5 mM | VMP5 & VMP6  0.625 μM | 1,802 | 492 |
|  | Sak 9 REV | ATTAGCGAACAAACCACTCGTC |  |  |  |  |
| IS.13 | Sak 10 FW | CATCAGGAAGCGAAAGAAGA | 2.5 mM | 86 & 87  0.420 μM | 1,683 | 373 |
|  | Sak 10 REV | GAGGCACCCAATGGAACAA |  |  |  |  |
| IS.14 | Sak 11 FW | CAATGAACAAGGAGTAAACCAA | 1.5 mM | VMP5 & VMP6  0.625 μM | 1,552 | 492 |
|  | Sak 11 REV | AAAACAGCCACGAAGCCAG |  |  |  |  |
| IS.15 | Sak 12 B FW | CCCAACTATCCTTCTTAGCCAGTA | 2.5 mM | 86 & 87  0.420 μM | 1,599 | 289 |
|  | Sak 12 B REV | CGGCGATTACGGTAACGAC |  |  |  |  |
| IS.16 | primers that yielded specific amplicons could not be designed | | | | | |
| IS.17 | Sak 14 FW | TATTCAACTGCTCCATAACGG | 2.5 mM | 86 & 87  0.420 μM | 1,636 | 326 |
|  | Sak 14 REV | TAATCAAGGAACTGGTGACTCTC |  |  |  |  |
| IS.18 | Sak 15 FW | ATTGCCAAAATCAGAGGTGCT | 3.5 mM | VMP5 & VMP6  0.625 μM | 1,664 | 356 |
|  | Sak 15 REV | GGTCGCTATCAGACGCTTCA |  |  |  |  |
| IS.19 | Sak 16 FW | GTGACCACCGACGCTGTAA | 5.0 mM | VMP5 & VMP6  0.625 μM | 1,838 | 528 |
|  | Sak 16 REV | ATTTCTGATAGTTCGCACTCTG |  |  |  |  |
| IS.20 | Sak 17 FW | GCTGTGCTGTAAGTAACTCCCC | 1.5 mM | VMP5 & VMP6  0.625 μM | 1,641 | 331 |
|  | Sak 17 REV | AAAGCGACTGTTGCCTGC |  |  |  |  |
| IS.21 | Sak 18 FW | TATTCGTGGGCTCAATGGAT | 1.5 mM | VMP5 & VMP6  0.625 μM | 1,596 | 286 |
|  | Sak 18 REV | ATCATCGCTTCAGTTCCTGTATC |  |  |  |  |
| IS.22 | Sak 19 FW | TTACGGTGAACCAGACAGATAAC | 1.5 mM | VMP5 & VMP6  0.625 μM | 1,559 | 245 |
|  | Sak 19 REV | GTAAACTTCTGACCATTGATGAATC |  |  |  |  |
| IS.23 | Sak 20 FW | AAGAAAGCGTGGCAAACAAA | 4.5 mM | VMP5 & VMP6  0.833 μM | 1,640 | 330 |
|  | Sak 20 REV | TGACTGGCGGAGTGTGACTAA |  |  |  |  |
| IS.24/25 | EDL 3&6 FW | TGAAAACATTATCCGACACACAT | 4.5 mM | VMP5 & VMP6  0.625 μM | 1,834 | 530 |
|  | EDL 3&6 REV | CATCAACAACACGAAGGGAGTA |  |  |  |  |
| Phage A | Phage B REV | GTCAATACTGCCATACGCTAAT | 3.5 mM | VMP5 & VMP6  0.625 μM | 584 | 0 |
|  | Phage 1.1 FW | ACTTACGGATTCCAGAGTGC |  |  |  |  |
| Phage B | Phage B REV | See above phage A |  |  | 387 | 0 |
|  | Phage 2.1 FW | TAAGTGAGGAAGTGATAGGAAGTG |  |  |  |  |
| IS.26 | primers that yielded specific amplicons could not be designed | | | | | |
| IS.27 | Sak 13 FW | TTCTTTGTCCTGATCTGCCC | 4.5 mM | VMP5 & VMP6  0.833 μM | 1,698 | 388 |
|  | EDL 18 REV | ATGTCGGATGTTGTTGGTGA |  |  |  |  |
| IS.28 | primers that yielded specific amplicons could not be designed | | | | | |
| IS.29 | EC 1 A FW | ATGCTTCGTTTCGTATCACACA | 1.5 mM | VMP5 & VMP6  0.625 μM | 1,780 | 470 |
|  | EC 1 REV | ATTTACTTCTATGACTGTCCACCACTC |  |  |  |  |
| IS.30 | EC 3 FW | CTTACGACGGTCCTCTCTGATTT | 3.5 mM | VMP6 & 86  0.625 μM | 1,525 | 215 |
|  | EC 3 REV | GCGGTGACGGAGACATACATC |  |  |  |  |
| IS.31 | EC 4.1 FW | AGTTTTATCAGGTTGTCTGTGCT | 1.5 mM | VMP5 & VMP6  0.625 μM | 2,003 | 693 |
|  | EC 4.2 REV | TTCAGTTATGTCGTGGTCGG |  |  |  |  |
| IS.32 | EC 8 FW | AGAGCCAATCAACAGCACACT | 2.5 mM | 86 & 87  0.625 μM | 1,900 | 590 |
|  | EC 8 REV | GCCTTATCCAGACCAGAAAGTTT |  |  |  |  |
| IS.33 | EC11 IS FW | CGCTCAGACAGTGAACCG | 1.5 mM | VMP5 & VMP6  0.420 μM | 1,335 | 0 |
|  | EC11 IS REV | CTCACCTGACACTGTGAACC |  |  |  |  |
| IS.34 | EC 13 FW | CCCAAATACGAAGTTGCTCAG | 1.5 mM | VMP5 & VMP6  0.625 μM | 1,761 | 451 |
|  | EC 13 REV | CGGTAAGACTCATATCAGTATCAGGT |  |  |  |  |
| IS.35 | EC14 IS REV | TTCTGGCGTCGTTCTTGAA | 3.5 mM | VMP5 & VMP6  0.625 μM | 1,697 | 0 |
|  | IS629 14 IS REV | GGCATCAACAGGGAGTACAG |  |  |  |  |
| IS.36 | primers that yielded specific amplicons could not be designed | | | | | |
| IS.37 | EC 17 FW | GACGACTCACTGACTTCACCG | 2.5 mM | 86 & 87  0.420 μM | 2,030 | 720 |
|  | EC 17 REV | AGGACTTGGGGATGTGGATAT |  |  |  |  |
| IS.38 | EC 19 FW | CATCTTATCGGTCATTTTTATCG | 5.0 mM | VMP5 & VMP6  0.420 μM | 2,221 | 636 |
|  | EC 19 REV | AGCCTCTTTCTTTGGCATCTT |  |  |  |  |
| IS.39 | gne 13 | CTGAAAGAAGACGCTGGCTA | 1.5 mM | VMP5 & VMP6  0.420 μM | 1,536 | 226 |
|  | gne 14 | CGGGTATTCCGACGATATAAA |  |  |  |  |
| IS.40 | P1 A FW | CCTTGGGGTTATCCACTTATC | 3.5 mM | VMP5 & VMP6  0.420 μM | 1,623 | 313 |
|  | P1 A REV | TCAGGCATACGCTGGTAACT |  |  |  |  |
| IS.41 | P2 A FW | AGAGATTATGCTGGCGAAAC | 3.5 mM | VMP5 & VMP6  0.420 μM | 1,383 | 300 |
|  | P2 A REV | ACCCTCCTGTAATATGGCTGT |  |  |  |  |
| IS.42 | P3 A FW | ATGTTGTGTTGTATCTCTGGACTG | 1.5 mM | VMP5 & VMP6  0.420 μM | 1,993 | 624 |
|  | P3 A REV | CACGGGAGACTTTATCTGACA |  |  |  |  |
| IS.43 | P4 A FW | CCTGACGGTTATCGGTGTAC | 3.5 mM | VMP5 & VMP6  0.420 μM | 1,533 | 0 |
|  | P4 A REV | TTACTTTCGTGAACAAAATAGTGAT |  |  |  |  |
| IS.44 | P1 A FW | Same as IS.40 | 3.5 mM | VMP5 & VMP6  0.420 μM | 2,100 | 800 |
|  | P1 A REV |  |  |  |  |  |
| IS.45 | TW 3A FW | TCCCAAATAGCGTTATCCAG | 1.5 mM | VMP5 & VMP6  0.625 μM | 1,880 | 570 |
|  | TW 3A REV | AAAACTTGTGGCAGAAGGAA |  |  |  |  |
| IS.46 | TW 15 FW | AGACCATTCTGACAATCCCA | 1.5 mM | VMP5 & VMP6  0.625 μM | 1762 | 452 |
|  | TW 15 REV | GAAGATCAAACACAGGCGTAT |  |  |  |  |
| IS.47 | primers that yielded specific amplicons could not be designed | | | | | |
| IS.48 | primers that yielded specific amplicons could not be designed | | | | | |
